# Analyzing the Fit of IRT Models With the Hausman Test

**DOI:** 10.3389/fpsyg.2020.00149

**Published:** 2020-02-11

**Authors:** Jochen Ranger, Sören Much

**Affiliations:** ^1^Institute of Psychology, Martin-Luther-University Halle-Wittenberg, Halle (Saale), Germany; ^2^Institute for Empirical Economic Research, Department of Statistics, Leipzig University, Leipzig, Germany

**Keywords:** item response theory, 2-PL model, model fit, item fit, Hausman test

## Abstract

In this manuscript, the applicability of the Hausman test to the evaluation of item response models is investigated. The Hausman test is a general test of model fit. The test assesses whether for a model in question the parameter estimates of two different estimators coincide. The test can be implemented for item response models by comparing the parameter estimates of the marginal maximum likelihood estimator with the corresponding parameter estimates of a limited information estimator. For a correctly specified item response model, the difference of the two estimates is normally distributed around zero. The Hausman test can be used for the evaluation of item fit and global model fit. The performance of the test is evaluated in a simulation study. The simulation study suggests that the implemented versions of the test adhere to the nominal Type-I error rate well in samples of 1000 test takers and more. The test is also capable to detect misspecified item characteristic functions, but lacks power to detect violations of the conditional independence assumption.

## Analyzing the Fit of IRT Models With the Hausman Test

Item response models are measurement models that allow inferring individual traits from responses given to the items of a standardized test. Core of item response models are precise assumptions about the relation between the traits and the response in a single item and the interrelation of the responses from different items. These assumptions then serve as a mathematical basis for deducing statements about a test taker’s traits from his/her responses. The correctness of such inferential statements depends crucially on the validity of the item response model. As the results of psychological assessment often have important consequences, one has to guarantee that the conclusions drawn about the test takers have a sound basis. Therefore, it is indispensable to check the adequacy of the chosen item response model and its assumptions carefully. Such a check requires a powerful test of model fit.

Several tests of model fit have been proposed in the past. A short overview over of the different tests is given in the following section. In doing so, the focus is mainly on the two-parameter logistic model. Nothing will be said about tests that were proposed exclusively for the Rasch model and cannot be used in general; for such tests see Glas and Verhelst (1995), Suaréz Falcón and Glas (2003), and Maydeu-Olivares and Montaño (2013). The review does also not cover the general approaches used in non-parametric item response theory (Sijtsma, 1998) or tests within the Bayesian framework (Sinharay, 2016). Tests of differential item functioning will also not be addressed (Magis et al., 2010). Having given this overview, an alternative test of model fit is proposed. The test is an implementation of the general specification test of Hausman (1978) and can be used to assess the fit of single items or the global fit of the model. The Hausman test has several attractive features. It is easy to implement in case an efficient and an inefficient estimator is available. The test statistic has a known asymptotic distribution. Due to its generality, the test can be used for models (e.g., multidimensional IRT models), for which other tests are hard to implement. Furthermore, the test does not require the grouping of the responses, which sometimes conceals misspecification. The test is also powerful (Hausman and Taylor, 1980). The performance of the test is investigated in a simulation study. The application of the Hausman test is illustrated with an empirical example.

## Tests of Model Fit

Overviews of tests of model fit for item response models have been given by Swaminathan et al. (2006), Mavridis et al. (2007), Maydeu-Olivares (2013), and Glas (2016). Due to space limitations, only a condensed summary will be given here. Tests for model fit can broadly be classified into omnibus tests that assess the global fit of a model and tests that assess the local fit of a model in single items or item pairs. Both types of tests will be addressed in the following.

The most obvious test for the global fit of an item response model is the χ^2^-test that compares the observed frequencies of the possible response patterns with the expected marginal frequencies that are implied by the model. In practice, the expected marginal frequencies are usually very small. This invalidates the application of the standard asymptotic distribution theory, according to which the sample distribution of the χ^2^-statistic should be a chi-square distribution. A solution to this problem consists in pooling cells (Bartholomew and Tzamourani, 1999), an approximation of the sample distribution via a parametric bootstrap (Tollenaar and Mooijaart, 2003) or in a modification of the χ^2^-statistic in order to improve its small sample behavior (Kraus, 2009). Alternatively, the problem of sparse tables can be dealt with the so called limited information tests of model fit. These tests are denoted as limited information tests as not the whole response pattern is considered, but only the cross tabulations of item pairs or item triples. For each cross tabulation, the limited information tests compare the observed cell frequencies quantifying the co-occurrence of specific responses to the frequencies implied by the item response model. This is done for all cross tabulations jointly. Popular limited information tests are the M_2_-test of Maydeu-Olivares and Joe (2005, 2006), the test proposed by Cai et al. (2006) and the test proposed by Reiser (2008); see also Cagnone (2012). Recent developments are the ${\text{M}}_{2}^{*}$-test of Cai and Hansen (2013) and the C_2_-test of Cai and Monroe (2014) for ordinal data. A limited information test of approximate model fit was proposed by Maydeu-Olivares and Joe (2014). The reduction of the response patterns to cross tabulations resembles the typical proceeding in structural equation modeling where the empirical covariance matrix and the theoretical covariance matrix are compared. When using the polychoric correlation matrix instead of the standard covariance matrix, the methods are also applicable to item response models. The fit of a specific item response model can then be tested similar to confirmatory factor models (e.g., Wirth and Edwards, 2007; Shi et al., 2018).

Limited information tests do not necessarily have to be limited to cross tabulations. In theory, one can use any arbitrary function of the responses for which its observed realization is compared to its expectation under the model. The statistical theory for these so called generalized residuals was presented by Haberman (2009) and Haberman and Sinharay (2013). A test that goes beyond the simple cell frequencies is, for example, the information matrix test. According to the Bartlett identities, the expected Hessian matrix equals the expectation of the outer product of the score vector in case the model is specified correctly (Chesher et al., 1999). The fit of the model can therefore be tested by comparing the equality of both versions of the observed information matrix as suggested by White (1982). The information matrix test has been implemented for the two-parameter logistic model by Ranger and Kuhn (2012).

An alternative approach to the evaluation of global model fit consists in comparing models. In doing so, the item response model in question is embedded into a more flexible model that is able to account for a specific form of model misspecification. The need for the higher flexibility is then tested with a likelihood ratio test or a score test. A score test of global model fit was implemented by Ranger and Kuhn (2012), who replaced the logit link function of the two-parameter logistic model with the more flexible link function proposed by Czado (1994). Alternatively, one could compare the two-parameter logistic model with the three-parameter logistic model or with a multidimensional model. As tests based on a model comparison probe for a specific form of misspecification, they have more power to detect this form of misspecification than the unspecific limited information tests.

Most of the tests for global model fit are complemented by a test for the local fit of the item response model. Such local tests of model fit assess the validity of the item characteristic function in single items, the validity of the local independence assumption in item pairs or item triples or the validity of the assumed distribution of the latent trait. The first tests of the item characteristic function in single items were the Q_1_-test of Yen (1981) and the test proposed by Bock (1972); see also McKinley and Mills (1985). These tests are similar in spirit to the popular Hosmer–Lemeshow test of logistic regression models (Hosmer and Lemeshow, 2000). Both tests require that the test takers are grouped into subgroups according to their trait level. For each subgroup defined by a typical trait level, the observed frequencies of the possible responses are compared to the expected frequencies that are implied by the item characteristic function. The similarity of the observed and the expected frequencies is then compared via a χ^2^-test or a likelihood ratio test. Unfortunately, the large sample distribution of the test statistic is unknown for the tests. The difficulty to derive the sample distribution is partly caused by the grouping of the test takers according to a trait estimate instead of the true trait level. One solution of this problem is to approximate the distribution via a parametric bootstrap as it is done in the ltm package of Rizopoulos (2006). Stone (2000) and Stone and Zhang (2003) accounted for the measurement error of the trait estimates by resorting to posterior expectations. Orlando and Thissen (2000, 2003) finally suggested conditioning on the observable sum score when comparing observed and expected frequencies. They proposed the popular S−X^2^-test statistic, whose distribution can be approximated by a chi-square distribution very well.

Alternatively, one can assess the validity of the item characteristic function in single items by comparing models. For that purpose, one has to embed the item characteristic function of a single item into a more flexible one. The need for the additional flexibility can then be tested item-wise via a score test or a likelihood ratio test. Such a test was proposed by Glas (1999) and Glas and Suárez Falcón (2003). In their test, the item characteristic function of the two-parameter logistic model is extended by allowing for different item parameters in groups defined by the sum score. The need for the extension is then tested with a score test. In similar spirit, Ranger and Kuhn (2012) implemented a score test that assessed the need for a more flexible link function in single items. Douglas and Cohen (2001) and Haberman et al. (2013) evaluate the congruency of the estimated item characteristic function with a non-parametric estimate. Although their approaches are primarily graphical checks of model fit, they also allow for formal tests of model fit.

In order to assess the conditional independence assumption locally, one usually focuses on single item pairs. Again, there are two general approaches to the assessment of local fit, one based on the analysis of cross tabulations and the other one based on model comparisons. Similar to the limited information tests of global fit, the corresponding tests of local fit compare the data and the model’s predictions with respect to the cross tabulation of two items. Chen and Thissen (1997) proposed a χ^2^-test that assesses whether the observed frequencies in single cross tabulations are equal to the frequencies implied by the model. The distribution of the test statistic, however, cannot be approximated by a chi-square distribution well. Drasgow et al. (1995) suggested adjusting the test statistic and also proposed a critical value for it, which they determined with Monte Carlo methods. Reiser (1996), Haberman and Sinharay (2013), and Liu and Maydeu-Olivares (2014) finally derived the large sample distribution of the difference between the observed and expected frequencies in single cross tabulations. Liu and Maydeu-Olivares (2012) generalized the S−X^2^-test of Orlando and Thissen (2003) to item pairs. For this test, the cross tabulation in an item pair are considered separately for subgroups defined by the total test score. A similar test was suggested by Ip (2001) who implemented a Mantel–Haenszel test of local fit.

An alternative to the limited information tests of local independence are tests based on model comparisons. For that purpose, the item response model in question is embedded into an augmented model that allows for specific forms of local dependency. The need for the extension is then tested. The score test of Glas (1999), for example, tests whether the item difficulty in one item depends on the response to another item. One can also test for local violations of conditional independence with the help of a bifactor logistic model that assumes an additional latent trait for a pair of items (Liu and Thissen, 2012) or by testing for omitted cross loadings (Falk and Monroe, 2018). This is similar to testing for correlated residuals in structural equation models, an approach, that has also been suggested for item response models (Edwards et al., 2018). Model comparisons do not have to be limited to such standard models. In fact, any model that allows for local dependencies – as for example the model of Ip (2002) – could be employed, although such models were not explicitly developed for the analysis of model fit and have not been used for this purpose so far. A new approach that can not be assigned to one of the two classes was suggested by Edwards et al. (2018), who evaluated how item parameters change when items are removed. Basis of this approach is the conjecture that local dependency inflates the item discrimination parameters of items from correlated item clusters.

The assumption about the distribution of the latent trait that is needed for marginal maximum likelihood estimation is rarely verified, probably because marginal maximum likelihood estimation is claimed to be robust against a misspecified trait distribution. Nevertheless, there are tests that can be used for this purpose. The limited information tests of Glas (2016) and Li and Cai (2018) test whether the observed distribution of the sum score corresponds to the distribution implied by the model. An alternative approach is to compare models, by testing the marginal item response model against an augmented one with a more flexible trait distribution. Such tests have been used in linear mixed models and could easily be adapted; see Caffo et al. (2007), Alonso et al. (2008) or Efendi et al. (2017) for examples.

This short overview illustrates that one already has several good tests of model fit at hand. The test for global fit of Maydeu-Olivares and Joe (2005) and the item specific test of Orlando and Thissen (2003) usually work very well. The tests of Glas (1999) and Ranger and Kuhn (2012) can also be recommended. Nevertheless, in this manuscript, a new test is proposed. The test is an implementation of the general specification test of Hausman (1978). The test has several advantageous properties. The test is easy to implement for the two-parameter logistic model (in contrast to the information matrix test, for example). The test rests on a sound asymptotic theory (in contrast to the Q_1_-test, for example). It provides a general framework for the test of global and item fit and can readily be generalized to polytomous or multidimensional models (in contrast to the S−X^2^-test, for example). Furthermore, the test does neither require estimates of the latent trait nor the grouping of test takers.

## The Specification Test of Hausman

In 1978, Hausman proposed a general specification test that is based on the following simple, but ingenious idea: In case a model is specified correctly, two different estimators of the model parameters should result in similar estimates. This can be stated more formally as follows. Denote by $\hat{\beta }$ the estimates from a first estimator of the model’s parameters. This first estimator is required to be consistent and asymptotically normally distributed. Denote by $\hat{\alpha }$ the estimates from a second estimator that is also consistent and asymptotically normally distributed. The idea of Hausman (1978) was to use the difference vector $\text{d}=\hat{\alpha }-\hat{\beta }$ as an indicator of model misspecification. In case of a correctly specified model, the distribution of the difference vector **d** converges to a multivariate normal distribution with expectation of zero. Large absolute values of **d** therefore indicate the misfit of the model. The covariance matrix **Σ**_d_ of the difference vector **d**, which is needed for a formal test of model fit, has a simple expression in case the first estimator is efficient and the second estimator is inefficient (that is, does not reach the Cramer-Rao bound). Under these conditions, the covariance matrix **Σ**_d_ is just the difference $\Sigma_{\hat{\alpha }}-\Sigma_{\hat{\beta }}$ of the covariance matrix of the inefficient estimator $\Sigma_{\hat{\alpha }}$ and the covariance matrix of the efficient estimator $\Sigma_{\hat{\beta }}$. This simple relation follows from the fact that the efficient estimator $\hat{\beta }$ is not correlated with the differences in **d**. The efficient estimator and the differences are uncorrelated as otherwise a linear combination of $\hat{\beta }$ and **d** would yield a consistent estimator with lower asymptotic variance (Hausman, 1978). The Hausman test can easily be implemented for item response models. For this purpose, we focus on the two-parameter logistic model, although a polytomous or a multidimensional item response model could be used as well.

The two-parameter logistic model has two parameters per item g, the intercept parameter β_0g_ and the discrimination parameter β_1g_. These item parameters are usually estimated with marginal maximum likelihood estimation by determining those parameter values that maximize the marginal likelihood of the observed response patterns in a calibration sample (Bock and Aitkin, 1981; Baker and Kim, 2004). The marginal maximum likelihood estimator is consistent, asymptotically efficient and normally distributed in the limit. Thus, the estimator can act as the first component of the Hausman test. Alternatively, the item parameters can be estimated via limited information estimation (Maydeu-Olivares and Joe, 2005). Limited information estimation is based on item pairs. For each item pair, the frequency of a positive response to both items is determined. The observed frequencies are stacked to vector **o**. Then, those values of the item parameters are determined that make the corresponding expected frequencies **e** as similar to the observed frequencies **o** as possible. In the simplest version, this boils down to choosing those parameter values that minimize the sum of the squared differences **(o−e)′(o−e)**. The limited information estimator is consistent and has a normal distribution in the limit (Maydeu-Olivares and Joe, 2005). Its asymptotic covariance matrix can be determined with the asymptotic covariance matrix of the observed frequencies and the delta method. In contrast to the marginal maximum likelihood estimator, the limited information estimator is not efficient. Hence, the limited information estimator can serve as the second component of the Hausman test. The Hausman test can be implemented as a test of item fit or as a test of model fit by selecting appropriate elements from the difference vector.

For the Hausman test of item fit, the items are analyzed separately. Denote by β^g=′(β^0⁢g,β^1⁢g) the marginal maximum likelihood estimates of the intercept parameter and the discrimination parameter in item g. Denote likewise by ${\hat{\alpha }}_{g}^{{}^{\prime }}=\left({\hat{\alpha }}_{0\,g},{\hat{\alpha }}_{1\,g}\right)$ the corresponding limited information estimates. As an item specific indicator of misspecification, we suggest using the difference ${\text{d}}_{g}={\hat{\alpha }}_{g}-{\hat{\beta }}_{g}$. This misspecification indicator is asymptotically distributed as a bivariate normal random variate with expectation of zero and covariance matrix $\Sigma_{d_{g}}=\Sigma_{{\hat{\alpha }}_{g}}-\Sigma_{{\hat{\beta }}_{g}}$ (Hausman, 1978). Here, the matrix $\Sigma_{{\hat{\beta }}_{g}}$denotes the covariance matrix of the marginal maximum likelihood estimator and the matrix $\Sigma_{{\hat{\alpha }}_{g}}$ the covariance matrix of the limited information estimator. These are the corresponding 2 × 2–submatrices that are taken from the covariance matrix of all item parameters. The fit of the item can be assessed with the test statistic

        
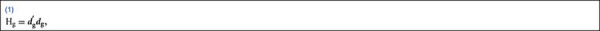


which should be small in case the model fits. Due to the bivariate normality of ***d***_g_, the asymptotic distribution of ***H***_g_ is a mixture of chi-square distributions. This follows from the fact that ${\text{d}}_{g}^{{}^{\prime }}\,{\text{d}}_{g}$ can be represented as $\sum_{i=1}^{2}\lambda_{i}\,z_{i}^{2}$, where the summands *z*_i_ are independent and standard normally distributed random variates and the coefficients λ_i_ the eigenvalues of **Σ**_d_g__ (Yuan and Bentler, 2010). This distribution can be approximated as follows: Denote by λ_1_ and λ_2_ the first and second eigenvalue of **Σ**_d_g__and define the quantities $a=\sum_{i=1}^{2}\lambda_{i}^{2}/\sum_{i=1}^{2}\lambda_{i}$ and $b={\left(\sum_{i=1}^{2}\lambda_{i}\right)}^{2}/\sum_{i=1}^{2}\lambda_{i}^{2}$. Then, the test statistic *H*_g_ is approximately distributed as

(2) $$H_{g}∼a\cdot \chi_{\text{b}}^{2},$$

where $\chi_{b}^{2}$ is a chi-square random variate with *b* degrees of freedom. This approximation is of wide use in categorical data analysis and structural equation modeling and dates back to Welch (1938). The transformation in Equation 2 equalizes the moments of the chi-square distribution to the moments of the test statistic; see Yuan and Bentler (2010) for more details. Using the approximation in Equation 2 instead of a standard Wald test has the advantage that one avoids inverting the covariance matrix **Σ**_d_g__, which is often close to singularity. The approximation is also simple to implement as the sum of the eigenvalues equals the sum of the diagonal elements of the covariance matrix.

The Hausman test can also be used to assess the global fit of the model. Denote by β^T=′(β^11,…,β^1⁢G) the marginal maximum likelihood estimates of the discrimination parameters in the G items of the test and by α^T=′(α^11,…,α^1⁢G) the corresponding limited information estimates. The global model fit can be tested with the difference vector ${\text{d}}_{T}={\hat{\alpha }}_{T}-{\hat{\beta }}_{T}$. The covariance matrix **Σ**_d_T__ of ***d***_T_ is the difference of the covariance matrices of the two estimators $\Sigma_{{\hat{\alpha }}_{T}}-\Sigma_{{\hat{\beta }}_{T}}$. These matrices are the g × g–submatrices of the discrimination parameters that are taken from the complete covariance matrix of all parameter estimates. The global fit of the model can be tested with the test statistic

        
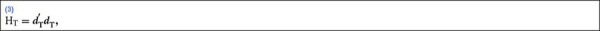


which is distributed as a mixture of chi-square distributions. The distribution can be approximated as in Equation 2. The global test of model fit is based on the discrimination parameters exclusively in order to avoid numerical problems. When including the intercept parameters, the test becomes numerically instable due to rank deficiencies of the covariance matrix **Σ**_d_T__.

When determining the covariance matrix **Σ**_d_g__ or **Σ**_d_T__of the misspecification indicator, one has to exercise caution that the resulting covariance matrix is positive definite. First, it is recommendable to evaluate the covariance matrices of the two estimators at the same parameter values, for example, the marginal maximum likelihood estimates (Ruud, 1984). Additionally, one has to use the expected information matrix and not the observed one when determining the covariance matrix of the marginal maximum likelihood estimator. Alternatively, one can determine the required covariance matrix **Σ**_d_g__ or **Σ**_d_T__ via a parametric bootstrap. For this purpose, one generates several bootstrap samples with the assumed model and the estimated item parameters. In each bootstrap sample, the parameters are estimated with the two estimators and the difference is calculated. The covariance matrix can then be estimated as the sample covariance matrix of the differences from the bootstrap samples. White (1982) proposed an alternative estimator of the covariance matrix that guarantees positive semi-definiteness, but this estimator requires additional implementation effort. Here, we focus on the versions that can be implemented with standard software and do not require advanced statistical skills.

## Simulation Study

In order to assess the performance of the proposed tests, two simulation studies were conducted. In the first simulation study, the focus was on the size of the tests. In the second simulation study, the focus was on their power.

### Simulation Study I

In the first simulation study, the size of the Hausman tests was investigated. Special attention was paid to its dependency on factors such as the length of the test, the size of the sample and the values of the item parameters. The simulation study consisted of three scenarios. In the first scenario, we explored the size of the tests for different values of the item parameters, but left the number of items and the size of the sample fixed. In the second scenario, we systematically varied the length of the test and the sample size. In the third scenario, we investigated the performance of the Hausman test in a multidimensional item response theory model. The simulation study was conducted with the statistical environment R (R Development Core Team, 2009). All scripts are available from the authors on request.

#### First Scenario: Effect of Parameter Values

In the first scenario, the data were generated for a test of 20 items and 1000 test takers according to the two-parameter logistic model. The item parameters of the test were determined by fully crossing five levels (−1.50, −0.75, 0.00, 0.75, 1.50) of the intercept parameter with four levels (0.6, 1.0, 1.4, 1.8) of the discrimination parameter. These values should cover the typical values the parameters have in real data. Having generated the data, the item parameters were estimated with the marginal maximum likelihood estimator via the package ltm (Rizopoulos, 2006). Then, the item parameters were re-estimated with the limited information estimator described in the previous section. This estimator was implemented in R by the authors. The item specific variant (H_g_-test) and the global variant (H_T_-test) of the Hausman test were performed. Both variants were implemented in two versions. In the first version, we determined the covariance matrix of the misspecification indicator by subtracting the covariance matrices of the two estimators; see the previous section. The covariance matrix of the marginal maximum likelihood estimator was determined by inverting the expected information matrix. The covariance matrix of the limited information estimator was determined as described by Maydeu-Olivares and Joe (2005). In the second version, we approximated the covariance matrix of the misspecification indicators by a parametric bootstrap. For that purpose, we generated 200 bootstrap samples with the two-parameter logistic model using the marginal maximum likelihood estimates. For each bootstrap sample, the misspecification indicator was calculated. The covariance matrix of the bootstrap estimates was then used as a proxy of the true covariance matrix. We included the bootstrap version as this version is easier to implement and therefore might be the preferred approach of practitioners. The sequence of data generation, parameter estimation and fit assessment was repeated 250 times.

The results for the first simulation scenario can be found in Table 1. There, the empirical rejection rates are reported for the two versions of the item specific variant of the Hausman test and different nominal Type-I error rates α. As no misspecification was present, the empirical rejection rates should be close to the nominal Type-I error rate.

**TABLE 1:** Empirical rejection rates of the item specific variant of the Hausman test (H_g_-test) for two versions (original/bootstrap), different combinations of item parameters (β_1g_/β_0g_) and different nominal type-I error rates α (0.10/0.05/0.01) in the first simulation scenario without misfit.

**β_1g_**	**β_0g_**	**Original**			**Bootstrap**		
		**α**			**α**		
		**0.10**	**0.05**	**0.01**	**0.10**	**0.05**	**0.01**
	–1.50	0.10	0.05	0.01	0.10	0.04	0.00
	–0.75	0.08	0.05	0.02	0.07	0.04	0.01
0.6	0.00	0.10	0.04	0.01	0.10	0.04	0.01
	0.75	0.11	0.06	0.02	0.09	0.06	0.02
	1.50	0.15	0.09	0.02	0.16	0.08	0.01

	–1.50	0.13	0.08	0.01	0.11	0.07	0.01
	–0.75	0.14	0.07	0.02	0.12	0.06	0.02
1.0	0.00	0.12	0.06	0.02	0.11	0.05	0.00
	0.75	0.15	0.09	0.01	0.16	0.07	0.01
	1.50	0.09	0.04	0.01	0.08	0.03	0.00

	–1.50	0.10	0.04	0.01	0.09	0.04	0.01
	–0.75	0.14	0.06	0.02	0.13	0.06	0.01
1.4	0.00	0.14	0.08	0.02	0.14	0.08	0.02
	0.75	0.11	0.08	0.02	0.10	0.06	0.01
	1.50	0.10	0.04	0.01	0.09	0.04	0.01

	–1.50	0.14	0.08	0.04	0.12	0.08	0.03
	–0.75	0.12	0.06	0.02	0.10	0.05	0.02
1.8	0.00	0.15	0.07	0.01	0.13	0.06	0.01
	0.75	0.15	0.06	0.03	0.11	0.06	0.03
	1.50	0.09	0.06	0.02	0.09	0.04	0.01

*Results based on 250 replications.*

The results in Table 1 suggest that the two versions of the item specific H_g_-test adhere to the nominal Type-I error rate well. An analysis of the relation between the values of the item parameters and the empirical rejection rates in Table 1 with a generalized estimating equation model did not reveal effects that have importance in practice. This implies that the good performance of the item specific tests is not bound to favorable combinations of the item parameters. The versions of the global H_T_-test were slightly too liberal. The empirical rejection rates of the original version were 0.14, 0.10 and 0.04 for nominal Type-I error rates α of 0.10, 0.05 and 0.01. The rejection rates of the bootstrap version were 0.10, 0.05 and 0.03.

#### Second Scenario: Effect of Test Length and Sample Size

In the second scenario, we explored the performance of the Hausman test in different lengths of the test and different sizes of the sample. We used the two-parameter logistic model for data generation and data analysis. The item parameters were determined by fully crossing two levels of the discrimination parameter (0.8, 1.2) with ten levels of the intercept parameter that were equally spaced between −1.5 and 1.5. Data were generated for 3 × 3 simulation conditions that were defined by fully crossing three sample sizes (250 subjects/1000 subjects/10000 subjects) with three lengths of the test (10 items/20 items/40 items). Sample sizes from 250 to 10000 subjects were considered as this range covers the typical sample sizes in practice. A length from 10 to 40 items was chosen as we regarded this as representative for psychological tests. Conditions with more items were not included (e.g., in order to simulate an application to item banks in adaptive testing) as already the limited study gave a clear picture of the effects. Besides, limited information estimation is computationally intensive in very long tests (>100 items). For each simulation condition, 250 simulation samples were analyzed. The results can be found in Table 2. There, the empirical rejection rates are given for the 3 × 3 simulation conditions and different nominal Type-I error rates α. As the empirical rejection rates did not depend on the values of the item parameters, the results have been averaged over the items. The empirical rejection rates should be close to the nominal Type-I error rates.

**TABLE 2:** Empirical rejection rates of the two versions (original/bootstrap) of the two Hausman test variants H_g_ and H_T_ for different sample sizes N, test lengths G and nominal type-I error rates α (0.10/0.05/0.01) in the second simulation scenario without misfit.

		**Original**						**Bootstrap**					
		**H_g_-test**			**H_T_-test**			**H_g_-test**			**H_T_-test**		
		**α**			**α**			**α**			**α**		
***G***	***N***	**0.10**	**0.05**	**0.01**	**0.10**	**0.05**	**0.01**	**0.10**	**0.05**	**0.01**	**0.10**	**0.05**	**0.01**
	250	0.21	0.14	0.06	0.35	0.28	0.18	0.08	0.05	0.02	0.11	0.07	0.04
10	1000	0.12	0.07	0.02	0.14	0.08	0.04	0.10	0.05	0.01	0.09	0.05	0.01
	10000	0.10	0.05	0.01	0.06	0.03	0.01	0.10	0.05	0.01	0.06	0.03	0.01

	250	0.17	0.10	0.04	0.39	0.32	0.20	0.09	0.05	0.01	0.11	0.08	0.02
20	1000	0.12	0.07	0.02	0.19	0.12	0.04	0.10	0.06	0.01	0.12	0.07	0.03
	10000	0.11	0.06	0.01	0.12	0.06	0.01	0.10	0.06	0.01	0.10	0.06	0.01

	250	0.16	0.10	0.03	0.52	0.42	0.26	0.10	0.05	0.01	0.14	0.08	0.04
40	1000	0.11	0.06	0.01	0.19	0.13	0.01	0.10	0.05	0.01	0.11	0.03	0.00
	10000	0.10	0.05	0.01	0.12	0.05	0.01	0.10	0.05	0.01	0.08	0.04	0.00

*Results based on 250 replications. Results for item specific tests have been averaged over the items. **H*_*g*_*-test: Hausman test of item fit; **H*_*T*_*-test: Hausman test of global fit.*

The findings in Table 2 suggest that the bootstrap versions of the two Hausman test variants adhere to the nominal Type-I error rate rather well, irrespective of the test length and the sample size. The global test tends to be slightly too liberal in small samples. The performance of the original version of the two Hausman test variants depends on the sample size. The item specific version already works well in moderate samples, but the global version of the test requires samples of at least 10000 subjects. In smaller samples, the tests are too liberal.

#### Third Scenario: Application to a Two-Dimensional Model

In the third scenario, we explored the performance of the Hausman test in a two-dimensional version of the two-parameter logistic model. The two-dimensional version relates the solution probability to the linear combination β_0g_ + β_1g_θ + β_2g_ω of two latent traits, θ and ω. The intercept parameters β_0g_ and the discrimination parameters β_1g_ of the first trait were set to the same values as in the second simulation scenario (see section “Second Scenario: Effect of Test Length and Sample Size”). The discrimination parameters β_2g_ of the second latent trait were set to zero in half of the items and to 0.8 or 1.2 in the remaining ones. Data were generated for a test of 20 items and a calibration sample of 1000 test takers. This condition was chosen in order to assess whether the minimal requirements of the Hausman test with respect to the sample size and the test length are similar for unidimensional and multidimensional models. Samples of 250 subjects were not considered as multidimensional models are difficult to fit with small samples. Having generated the data, the item parameters of the two-dimensional model were estimated with the marginal maximum likelihood estimator and the limited information estimator. The discrimination parameter of the first item and the second latent trait (β_21_) was fixed to zero in order to identify the model. Having fit the model, the two variants of the Hausman test were performed. The first variant was the item specific H_g_-test based on the item parameters of a single item. For this test, the misspecification indicator was formed by subtracting the different estimates of the item parameters. The second variant was the global H_T_-test. For this test, the misspecification indicator was formed by subtracting the different estimates of all 2 × 20 discrimination parameters. Only the original versions of the tests were performed. The bootstrap versions of the tests were not implemented as estimating the two-dimensional model was computationally too expensive. Altogether, 250 simulation samples were analyzed. The results can be found in Table 3. Table 3 contains the empirical rejection rates of the two test variants for different nominal Type-I error rates α. Note that the results of the item specific tests have been averaged over the items. The findings in Table 3 illustrate that the tests adhere to the nominal Type-I error rate rather well and imply that the Hausman test can be extended to multidimensional models.

**TABLE 3:** Empirical rejection rates p of the two variants (H_g_-test/H_T_-test) of the Hausman test for different nominal type-I error rates α (0.10/0.05/0.01) in the third simulation scenario without misfit.

	**H_g_-test**			**H_T_-test**		
**α**	0.10	0.05	0.01	0.10	0.05	0.01

*p*	0.09	0.05	0.02	0.08	0.05	0.03

*Results based on 250 replications. Item specific results have been averaged over the items. **H*_*g*_-*test: Hausman test of item fit; **H*_*T*_*-test: Hausman test of global fit.*

### Simulation Study II

In the second simulation study, the power of the Hausman tests to detect several forms of misspecification was explored. The proceeding was similar to Orlando and Thissen (2003) and Ranger and Kuhn (2012). The study consisted of three scenarios with different forms of misspecification. In all scenarios, the data were analyzed as in the first simulation study on the size of the Hausman test (see section “Simulation Study I”). All scripts that are necessary to reproduce the study are available from the authors on request.

In addition to the different Hausman tests, several alternative tests of local and global fit were performed. The first alternative test of local fit was the χ^2^-test of item fit provided by the package ltm (Rizopoulos, 2006). This test is similar to the Q_1_-test of Yen (1981). The test takers are grouped according to their estimated trait level. The observed responses in each group are then compared to the expected responses via a χ^2^-statistic. The distribution of the test statistic is determined by a parametric bootstrap. The second test of local fit was the S−X^2^-test of item fit suggested by Orlando and Thissen (2003) that is implemented in the package mirt (Chalmers, 2012). The third test of local fit was the score test proposed by Ranger and Kuhn (2012). In this test, the item characteristic function of the two-parameter logistic model is embedded into a more flexible one with two additional parameters that modify its shape in the left and right tail. Setting both parameters to zero results in the item characteristic function of the two-parameter logistic model. The test was implemented by the authors. The global fit of the model was analyzed with the M_2_-test of Maydeu-Olivares and Joe (2005); for a description of this test see the overview given in the introduction. The M_2_-test is part of the package mirt (Chalmers, 2012).

#### First Scenario: Detection of Misspecified Item Characteristic Functions

The first scenario was concerned with the power of the Hausman tests to detect a misspecification of the item characteristic function. Simulation data sets were generated for a test with 20 items. As in the second scenario of the first simulation study (see section “Second Scenario: Effect of Test Length and Sample Size”), we used a two-parameter logistic model with discrimination parameters of 0.8 or 1.2 and intercept parameters equally spaced between −1.5 to 1.5. In four items, the item characteristic function of the two-parameter logistic model was replaced by an alternative item characteristic function. The alternative item characteristic functions were identical to the ones used in the study of Orlando and Thissen (2003) and Ranger and Kuhn (2012). In a first simulation condition (Condition ICC 1), the responses to the four misspecified items were generated according to the item characteristic function

(4) $$\text{P}\left(x_{\text{g}}=1|\theta \right)=\frac{c_{g}}{1+\exp\left[a_{g}\,\left(\theta -\left(b_{g}-l_{g}\right)\right)\right]}+\frac{1}{\exp\left[-a_{g}\,\left(\theta -b_{g}\right)\right]},$$

where the item parameters had the values of *a*_g_ = 4.25, *b*_g_ = 1.00, *c*_g_ = 0.25 and *l*_g_ = 1.50. The item characteristic function decreases from 0.25 to 0.04 for θ ∈ (−∞,0], but then increases monotonously to 1 for θ ∈ (0,∞) according to an S-shape. Such a function could, for example, reflect that moderately gifted test takers are seduced to choose a wrong distractor that taps half knowledge. As the fundamental assumption of monotonicity is violated, this violation should be easy to detect.

In a second simulation condition (Condition ICC 2), the four misspecified items had the item characteristic function of the four-parameter logistic model

(5) $$\text{P}\left(x_{\text{g}}=1|\theta \right)=c_{g}+\left(1-c_{g}\right)\cdot l_{g}\cdot \frac{1}{\exp\left[-a_{g}\,\left(\theta -b_{g}\right)\right]},$$

with item parameter values of *a*_g_ = 3.40, *b*_g_ = 0.50, *c*_g_ = 0.00 and *l*_g_ = 0.70. The item characteristic function is S-shaped, with a lower asymptote of 0.00 and an upper asymptote of 0.70. Such an item characteristic function accounts for the loss of concentration or careless mistakes. As the four-parameter logistic model can be approximated by the two-parameter logistic model quite well, this form of misspecification is difficult to detect.

Data were generated for samples with 250 and 1000 subjects. Samples with 10000 subjects were not considered anymore. Fully crossing both factors (2 sample sizes × 2 forms of misspecification) defined four simulation conditions. For each simulation condition, we generated 250 simulation samples. Each simulation sample was analyzed as in the first simulation study on the size of the Hausman test (see section “Simulation Study I”). Additionally, the alternative tests of model fit were performed. The empirical rejection rates of the tests can be found in Table 4 for different nominal Type-I error rates α. In Table 4, we distinguish between the items with correctly specified item characteristic function, where the rejection rate should be close to the nominal Type-I error rate, and the items with misspecified item characteristic function, where the rejection rate should be as high as possible.

**TABLE 4:** Empirical rejection rates of several tests of model fit for different sample sizes N and different nominal type-I error rates α (0.10/0.05/0.01) in the first simulation scenario where the item characteristic function was misspecified in some items.

	**Global fit**											
**ICC**	**ICC 1**						**ICC 2**					
***N***	**250**			**1000**			**250**			**1000**		
**α**	**0.10**	**0.05**	**0.01**	**0.10**	**0.05**	**0.01**	**0.10**	**0.05**	**0.01**	**0.10**	**0.05**	**0.01**

H_T_-test (O)	1.00	1.00	1.00	1.00	1.00	1.00	0.16	0.09	0.04	0.53	0.39	0.18
H_T_-test (B)	0.98	0.97	0.95	1.00	1.00	1.00	0.04	0.02	0.00	0.47	0.30	0.13
M_2_-test	0.94	0.90	0.78	1.00	1.00	1.00	0.12	0.05	0.01	0.10	0.04	0.00

	**Item Fit: Correctly specified items**											

**ICC**	**ICC 1**						**ICC 2**					
***N***	**250**			**1000**			**250**			**1000**		
**α**	**0.10**	**0.05**	**0.01**	**0.10**	**0.05**	**0.01**	**0.10**	**0.05**	**0.01**	**0.10**	**0.05**	**0.01**

H_g_-test (O)	0.40	0.30	0.16	0.62	0.54	0.40	0.13	0.08	0.02	0.14	0.08	0.02
H_g_-test (B)	0.27	0.18	0.07	0.60	0.52	0.38	0.08	0.04	0.01	0.13	0.07	0.02
S−X^2^-test	0.13	0.06	0.01	0.27	0.17	0.06	0.10	0.05	0.01	0.11	0.05	0.01
χ^2^-test	0.16	0.09	0.02	0.30	0.20	0.07	0.10	0.05	0.01	0.12	0.06	0.01
SC-test	0.12	0.07	0.02	0.14	0.08	0.03	0.09	0.04	0.01	0.08	0.04	0.01

	**Item Fit: Misspecified items**											

**ICC**	**ICC 1**						**ICC 2**					
***N***	**250**			**1000**			**250**			**1000**		
**α**	**0.10**	**0.05**	**0.01**	**0.10**	**0.05**	**0.01**	**0.10**	**0.05**	**0.01**	**0.10**	**0.05**	**0.01**

H_g_-test (O)	1.00	0.99	0.98	1.00	1.00	1.00	0.13	0.06	0.02	0.33	0.20	0.05
H_g_-test (B)	0.99	0.98	0.94	1.00	1.00	1.00	0.06	0.02	0.00	0.30	0.18	0.05
S−X^2^-test	0.87	0.80	0.61	1.00	1.00	1.00	0.19	0.11	0.04	0.44	0.31	0.14
χ^2^-test	0.85	0.76	0.51	1.00	1.00	0.99	0.11	0.04	0.01	0.25	0.14	0.04
SC-test	1.00	0.99	0.96	1.00	1.00	1.00	0.35	0.25	0.11	0.86	0.78	0.57

*Results based on 250 replications. Results for item specific tests have been averaged over the items. **H*_*T*_*-test: Hausman test of global fit (O: original/B: bootstrap); *M*_2_-test: test of Maydeu-Olivares and Joe (2005); **H*_*g*_*-test: Hausman test of item fit (O: original/B: bootstrap); *S*−*X*^2^-test: test of Orlando and Thissen (2003); χ^2^-test of Rizopoulos (2006); SC-test: score test of Ranger and Kuhn (2012); ICC 1: Non-monotone item characteristic function, ICC 2: bounded item characteristic function.*

The findings in Table 4 suggest that misspecification caused by the non-monotone item characteristic function (ICC 1) is easy to detect. The global tests of model fit all have a rejection rate near 1.00. The item specific tests are capable to detect the misspecified items with high probability. The score test and the two versions of the Hausman test have the highest rejection rates in the affected items. The item specific variants of the Hausman tests, however, also have a rather high rate of false alarms in the items without misspecification. The original version of the Hausman test is worst in this respect, probably because the test is liberal. The score test, on the other hand, has a very low rate of false alarms. Misspecification in the form of an upper boundary (ICC 2) is much harder to detect. The global variants of the Hausman test, for example, detect the misspecification in only half of the samples with 1000 subjects. The M_2_-test has no power at all. The item specific test with the highest power is again the score test. This test also has a very low rate of false alarms. The other tests of item fit clearly fall behind.

#### Second Scenario: Detection of Local Dependencies

In the second scenario, the power of the Hausman test to detect local violations of the conditional independence assumption was investigated. The process of data generation was similar to the one used in the first simulation scenario (see section “First Scenario: Detection of Misspecified Item Characteristic Functions”). That is, the responses were generated for a test of 20 items with the two-parameter logistic model. The same item parameters were used as before. When generating the data, the conditional independence assumption was violated locally. This was achieved by coupling the responses in single item pairs via a bivariate normal copula with a copula parameter of ρ = 0.50 (Joe, 1997). This limits the misspecification of the model to the conditional independence assumption as the copula will not affect the item characteristic function. Conditional independence was violated in 10 of the 190 item pairs, namely in pair (1–2), (4–5), (6–7), (9–10), (11–12), (14–15), (16–17) and (19–20). Local violations of the conditional independence assumption occur, for example, in case some items share the same content or depend on the same knowledge. Two simulation conditions were considered that were defined by the sample size (250 subjects/1000 subjects). For each condition, 250 samples were generated. The data were analyzed as before. In addition to the two variants of the Hausman test, the alternative tests of model fit were performed; see section “First Scenario: Detection of Misspecified Item Characteristic Functions.” The results can be found in Table 5 for different nominal Type-I error rates α. The results for the item specific tests are reported separately for the correctly specified and the misspecified items with local dependencies.

**TABLE 5:** Empirical rejection rates of several tests of model fit for different sample sizes N and different nominal type-I error rates α (0.10/0.05/0.01) in the second simulation scenario with local dependencies in some item pairs.

	**Global fit**					
***N***	**250**			**1000**		
**α**	**0.10**	**0.05**	**0.01**	**0.10**	**0.05**	**0.01**

H_T_-test (O)	0.90	0.87	0.78	1.00	1.00	1.00
H_T_-test (B)	0.67	0.58	0.48	1.00	1.00	1.00
M_2_-test	1.00	1.00	1.00	1.00	1.00	1.00

	**Item fit: correctly specified items**					

***N***	**250**			**1000**		
**α**	**0.10**	**0.05**	**0.01**	**0.10**	**0.05**	**0.01**

H_g_-test (O)	0.21	0.13	0.05	0.22	0.14	0.06
H_g_-test (B)	0.13	0.07	0.02	0.20	0.13	0.05
S−X^2^-test	0.10	0.05	0.01	0.08	0.04	0.00
χ^2^-test	0.09	0.05	0.01	0.08	0.04	0.00
SC-test	0.10	0.05	0.01	0.10	0.05	0.01

	**Item fit: misspecified items**					
***N***	**250**			**1000**		
**α**	**0.10**	**0.05**	**0.01**	**0.10**	**0.05**	**0.01**

H_g_-test (O)	0.41	0.33	0.21	0.52	0.45	0.32
H_g_-test (B)	0.31	0.24	0.12	0.51	0.43	0.30
S−X^2^-test	0.10	0.05	0.01	0.10	0.06	0.01
χ^2^-test	0.10	0.05	0.01	0.11	0.06	0.01
SC-test	0.11	0.05	0.01	0.13	0.07	0.02

*Results based on 250 replications. Results for item specific tests have been averaged over the items. **H*_*T*_*-test: Hausman test of global fit (O: original/B: bootstrap); *M*_2_-test: test of Maydeu-Olivares and Joe (2005); **H*_*g*_*-test: Hausman test of item fit (O: original/B: bootstrap); *S*−*X*^2^-test: test of Orlando and Thissen (2003); χ^2^-test of Rizopoulos (2006); SC-test: score test of Ranger and Kuhn (2012).*

Table 5 corroborates that the global tests of model fit are capable to detect the additional association with high probability. The M_2_-test of Maydeu-Olivares and Joe (2005) performs best, a fact that is hardly surprising when one considers how the test is constructed. The item specific variants of the Hausman test are moderately successful in identifying the affected items. The Hausman tests, on the other hand, also have an elevated rate of false alarms in the correctly specified items such that the separation of correctly specified items from misspecified items is not easy. The alternative tests of item fit have no power to detect the local dependencies at all.

#### Third Scenario: Application to Multidimensional Models

The third simulation scenario was a replication of the first simulation scenario (misspecification of the item characteristic function) with a two-dimensional item response model. The model used for data generation and data analysis was the two-dimensional version of the two-parameter logistic model described in section “Third Scenario: Application to a Two-Dimensional Model.” Misspecification was created in four items by replacing the logit link between the response probability and the linear predictor with an alternative link function. In the first simulation condition (Condition ICC 1), the misspecification was of the form given in Equation 4, whereby the linear predictor *a*_g_(θ−*b*_g_) was replaced by the two-dimensional version β_0g_ + β_1g_θ + β_2g_ω. In the second simulation condition (Condition ICC 2), the response probability was bounded as in Equation 5. In doing so, the one-dimensional linear predictor was replaced by the corresponding two-dimensional linear predictor. Data were generated for a test of 20 items and a sample of 1000 subjects. Samples with 250 subjects were not considered anymore, as the two estimators (marginal maximum likelihood estimator/limited information estimator) have difficulties to converge in small samples. Having estimated the item parameters, the multidimensional variants of the Hausman test (see section “Third Scenario: Application to a Two-Dimensional Model”) were performed as well as the alternative tests. Bootstrap versions of the Hausman tests were not implemented as this was computationally too demanding. We also did not perform the χ^2^-test of Rizopoulos (2006) as this test has not been implemented for two-dimensional models. The empirical rejection rates of the tests can be found in Table 6 for different nominal Type-I error rates α. Again, the results are presented separately for items that are affected by misspecification and items that are not.

**TABLE 6:** Empirical rejection rates of several tests of item fit for different nominal type-I error rates α (0.10/0.05/0.01) in the third simulation scenario where the item characteristic function was misspecified in some items.

	**Global fit**					
**ICC**	**ICC 1**			**ICC 2**		
**α**	**0.10**	**0.05**	**0.01**	**0.10**	**0.05**	**0.01**

H_T_-test	0.34	0.24	0.10	0.16	0.08	0.03
M_2_-test	0.32	0.20	0.07	0.16	0.12	0.02

	**Item fit: correctly specified items**					

**ICC**	**ICC 1**			**ICC 2**		
**α**	**0.10**	**0.05**	**0.01**	**0.10**	**0.05**	**0.01**

H_g_-test	0.17	0.10	0.04	0.12	0.07	0.03
S−X^2^-test	0.17	0.09	0.02	0.13	0.07	0.02
SC-test	0.11	0.06	0.01	0.10	0.05	0.01

	**Item fit: misspecified items**					

**ICC**	**ICC 1**			**ICC 2**		
**α**	**0.10**	**0.05**	**0.01**	**0.10**	**0.05**	**0.01**

H_g_-test	0.29	0.21	0.10	0.19	0.12	0.04
S−X^2^-test	0.72	0.68	0.60	0.42	0.30	0.14
SC-test	0.96	0.94	0.88	0.72	0.66	0.48

*Results based on 250 replications. Item specific results have been averaged over the items. **H*_*T*_-*test: Hausman test of global fit; *M*_2_-test: test of Maydeu-Olivares and Joe (2005); **H*_*g*_-*test: Hausman test of item fit; *S*−*X*^2^-test: test of Orlando and Thissen (2003); SC-test: score test of Ranger and Kuhn (2012); ICC 1: non-monotone item characteristic function; ICC 2: bounded item characteristic function.*

The findings in Table 6 suggest that in comparison to the first scenario, the power of the global tests is lower. The global tests have low rejection rates especially in the case of a bounded item characteristic function. The item specific variant of the Hausman test also has little power. The score test performs best and is capable to detect the affected items with high probability without having an elevated rate of false alarms.

## Empirical Application

In addition to the simulation study, we compared the different tests in a real data set. For this purpose, we analyzed the Scored data set provided by the package irtoys (Partchev, 2014). The data set contains the real-life responses of 472 subjects to 18 multiple choice items scored as true or false. As the irtoys package has been intended for those teaching or learning item response theory, the data should be in close agreement with the two-parameter logistic model. The data were analyzed as follows. First, we fitted the two-parameter logistic model to the data using the marginal maximum likelihood estimator and the limited information estimator. Then, we performed all tests considered in the second simulation study. Neither the M_2_-test of global model fit (*M*_2_ = 134.5, *df* = 135, *p* = 0.49), nor the original version of the global Hausman test (*H*_T_ = 0.28, *p* = 0.14) or its bootstrap version (*H*_T_ = 0.28, *p* = 0.37) did reveal any signs of misspecification. Given the results from the second simulation study, this excludes local dependencies and grossly misspecified item characteristic functions. However, as global tests of model fit sometimes have low power to detect local misspecifications, we also performed the tests of item fit. The *p*-values of the different tests in the 18 items can be found in Table 7. All *p*-values lower than 0.05 have been highlighted.

**TABLE 7:** Overview over *p*-values of several tests of item fit in the 18 items of the scored data.

**Item**	**H_g_-test (O)**	**H_g_-test (B)**	**χ^2^-test**	**SC-test**	**S−X^2^-test**
1	0.09	0.12	0.82	0.69	0.84
2	0.22	0.24	0.85	0.36	0.37
3	0.89	0.91	0.31	**0.01**	0.35
4	0.53	0.56	0.72	0.27	0.66
5	0.78	0.81	0.91	0.94	0.95
6	0.06	0.10	0.05	0.69	0.21
7	0.59	0.61	0.25	0.74	0.96
8	0.46	0.53	0.34	0.93	0.82
9	0.32	0.43	0.25	0.67	0.88
10	0.93	0.94	0.55	0.33	0.21
11	0.57	0.62	0.05	0.12	**0.01**
12	0.87	0.87	0.66	0.82	0.93
13	0.90	0.92	**0.02**	0.63	0.07
14	0.21	0.30	0.90	0.17	0.51
15	0.42	0.49	0.85	0.11	0.24
16	**0.00**	**0.01**	0.64	**0.01**	0.18
17	0.28	0.36	0.19	0.93	1.00
18	0.25	0.31	0.10	0.24	0.29

**p-*values lower than α = 0.05 are highlighted. **H*_*g*_-*test: Hausman test of item fit (O: original/B: bootstrap); χ^2^-test of Rizopoulos (2006); SC-test: score test of Ranger and Kuhn (2012); *S*−*X*^2^-test: test of Orlando and Thissen (2003).*

In general, the results of the different tests agree insofar as most of the items do not seem to be in conflict with the two-parameter logistic model. There are, however, differences with respect to which items are flagged. The score test flags item 3 and item 16. Item 16 is also flagged by the two versions of the Hausman test. Somewhat surprising, the item is not flagged by the χ^2^-test and the S−X^2^-test, which on the other hand flag item 11 and 13. In order to identify the reasons for the different results, we estimated the item characteristic functions non-parametrically with the package KernSmoothIRT (Mazza et al., 2014). This analysis suggested that the item characteristic functions of item 3 and item 16 have a lower boundary of about 0.2. The item characteristic function of item 13 on the other hand seems to have some non-monotonicity around an average trait level of 0. The score test is by construction capable to detect deviations in the tail area. This might explain the significant results in item 3 and 16 and the insignificant result in item 13. The χ^2^-test and the S−X^2^-test on the other hand might be less sensitive to deviations in the tail area. The tests require the grouping of the data, either into trait groups or into groups defined by the sum score. In case the grouping is too coarse or the number of subjects in the extreme groups is rather small, the power to detect misspecifications in the tails might be low. This speculation is supported by the observation that increasing the number of groups from the default value of 10 to 14 reduced the p-value of the χ^2^-test in item 16 from 0.64 to 0.06. The Hausman test of item fit might also be more sensitive to a misspecification in the tail area than to misspecifications in the center as this probably affects the parameter estimates to a larger extent.

## Discussion

The analysis of model fit is a necessary prerequisite for the application of an item response model. According to Standard 3.9 of the Standards for Educational and Psychological Testing, evidence has to be given for the adequateness of an item response model before it can be used for psychological assessment (Sinharay and Haberman, 2014). This requires the application of tests that evaluate the fit of the model on a global level and a local level. Several such tests have been proposed since the beginning of item response modeling. Nowadays test takers can choose between tests that closely adhere to the nominal Type-I error rate and have high power to detect model misspecifications. Nevertheless, there is still need for further research in this area. Not all tests that work well in simple item response models can be generalized to polytomous or multidimensional models or to tests that mix categorical and continuous response formats. Other tests are computationally intensive and hard to implement in long scales with many response options. And some tests of global fit cannot be implemented as tests of item fit that can be used for item selection.

In a seminal paper, Hausman (1978) proposed a general specification test. The test is not bound to a specific statistical model and can be applied quite generally, among other things to item response models. The Hausman test has several attractive features. It is easy to implement in case an efficient and an inefficient estimator is available. Due to its generality, it can be used for models where the standard tests are hard to implement. Models for mixed response formats or the recently developed models for responses and response times (e.g., van der Linden, 2007) can be mentioned here. The Hausman test does not require the grouping of the data, which sometimes conceals misspecification. The test is also powerful provided that the parameter estimates of the different estimation approaches differ (Hausman and Taylor, 1980; White, 1982).

Despite its virtues, the Hausman test has never been implemented for item response models. This might be due to numerical difficulties that are caused by covariance matrices near singularity. In this manuscript, a novel implementation of the test was proposed that avoids these complications. The implementation can be used in order to test the global fit or the item specific fit. The performance of the implementations was investigated in a simulation study. The simulation study revealed that the tests adhere to the nominal Type-I error rate in samples of 1000 subjects and more. In smaller samples it is recommendable to resort to a bootstrap version of the tests. The simulation study also indicated that the tests have power to detect some, but not all forms of misspecification. The global variant of the Hausman test was on par with the M_2_-test in misspecified item characteristic functions in both a unidimensional and a multidimensional model, but had little power to detect local dependencies. The item specific variant was inferior to the score test, but similar to the S−X^2^-test when misspecified item characteristic functions had to be detected, at least in the one-dimensional two-parameter logistic model. It had the highest power to detect local dependencies. This suggests that the Hausman test is useful. The findings also imply that using several tests provides more informative about the exact cause of misspecification than the single tests on their own.

The simulation study was limited in scope, as every simulation study has to be. We did not analyze the performance of the test under unfavorable conditions, such as conditions with a considerable proportion of missing data. We did, however, consider conditions that are known to cause problems for tests of model fit, namely long tests and small discrimination coefficients (Shi et al., 2018). The performance of the Hausman test should also be analyzed for the three-parameter logistic model where some tests of model fit are known to become erratic (Chon et al., 2010) or for cognitive diagnosis models (Hu et al., 2016). More simulation studies are needed to get a better picture of the performance of the Hausman test.

More work could also be invested in the improvement of the small sample behavior of the test. Using the alternative estimate of the covariance matrix proposed by White (1982) might be a point to start. Alternatively, one could derive the covariance matrix within the estimation framework outlined by Maydeu-Olivares and Joe (2005). Equation 15 in their paper would be the point to start. This is topic of future research.

## Data Availability Statement

The raw data supporting the conclusions of this article will be made available by the authors, without undue reservation, to any qualified researcher.

## Author Contributions

All authors listed have made a substantial, direct and intellectual contribution to the work, and approved it for publication.

## Conflict of Interest

The authors declare that the research was conducted in the absence of any commercial or financial relationships that could be construed as a potential conflict of interest.
